# Supplementary material for: Habitat and landscape factors influence pollinators in a tropical megacity, Bangkok, Thailand
Source: PeerJ. 2018 Jul 20;6:e5335. doi: 10.7717/peerj.5335 (PMC6055598; doi:10.7717/peerj.5335)
Supplement: Supplemental Information 1 — Supplemental Figure S1a. A map of Bangkok, Thailand, showing all 52 green areas used in the study. Supplemental Figure S1b. Representative samples of how we measured percent vegetation surrounding each plot within 5 concentric circles (yellow, 100 m; green, 350 m; purple, 650 m; blue, 1,050 m; and pink 1,550 m radiuses). Top to bottom: (A) Suan Wachirabenchatat, (B) Wat Pathum Wanaram and Suan Lumpini, (C) Suan Sri Nakhon Khuean Khan (left) and School Sumran Wittaya (right), and (D) Dusit Zoo and Dusit Golf Course. [file peerj-06-5335-s001.pdf]

Habitat and landscape factors influence pollinators in a tropical megacity, Bangkok, Thailand

Supplemental Figure S1. Maps of the study area.

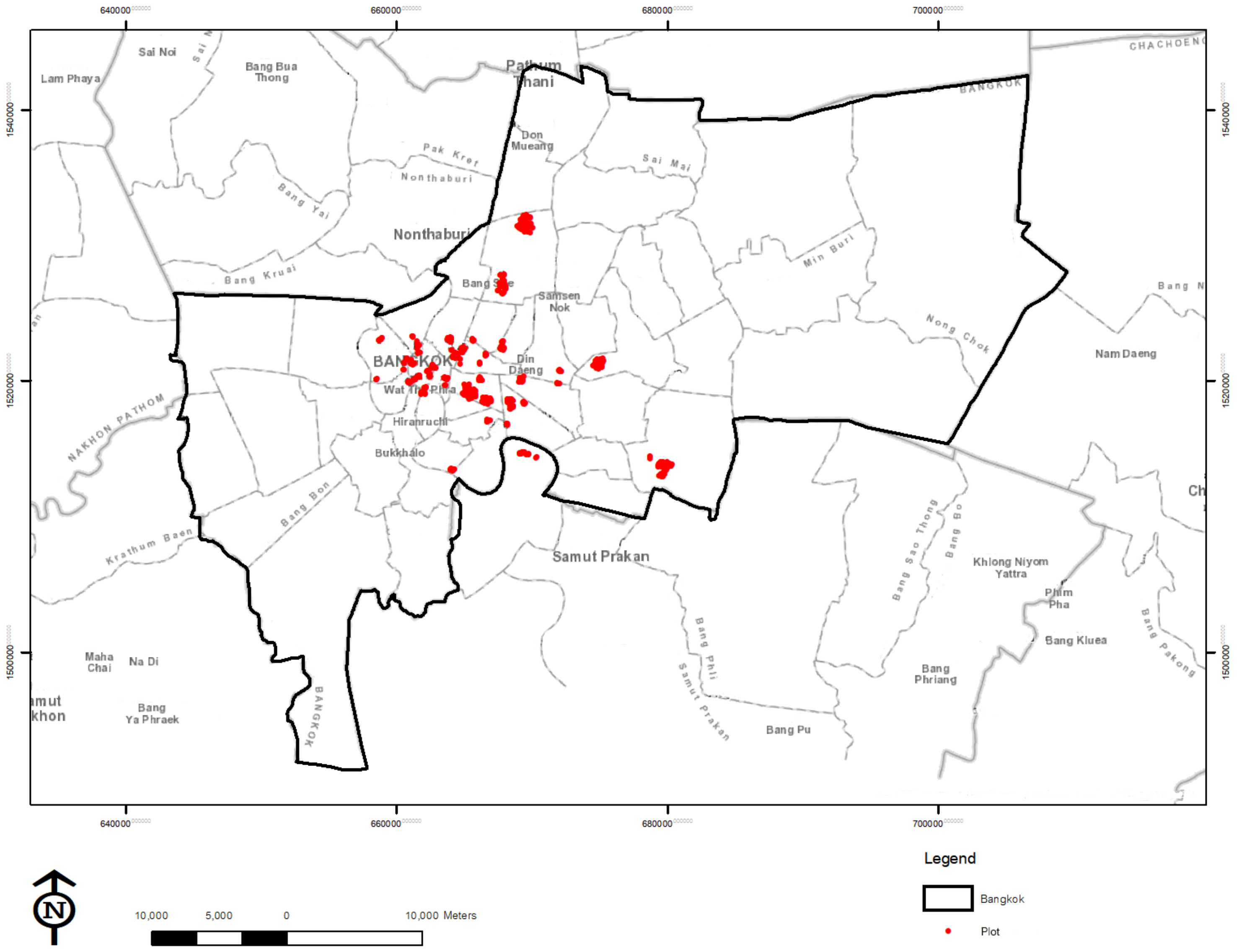

Supplemental Figure S1a. A map of Bangkok, Thailand, showing all 52 green areas used in the study.

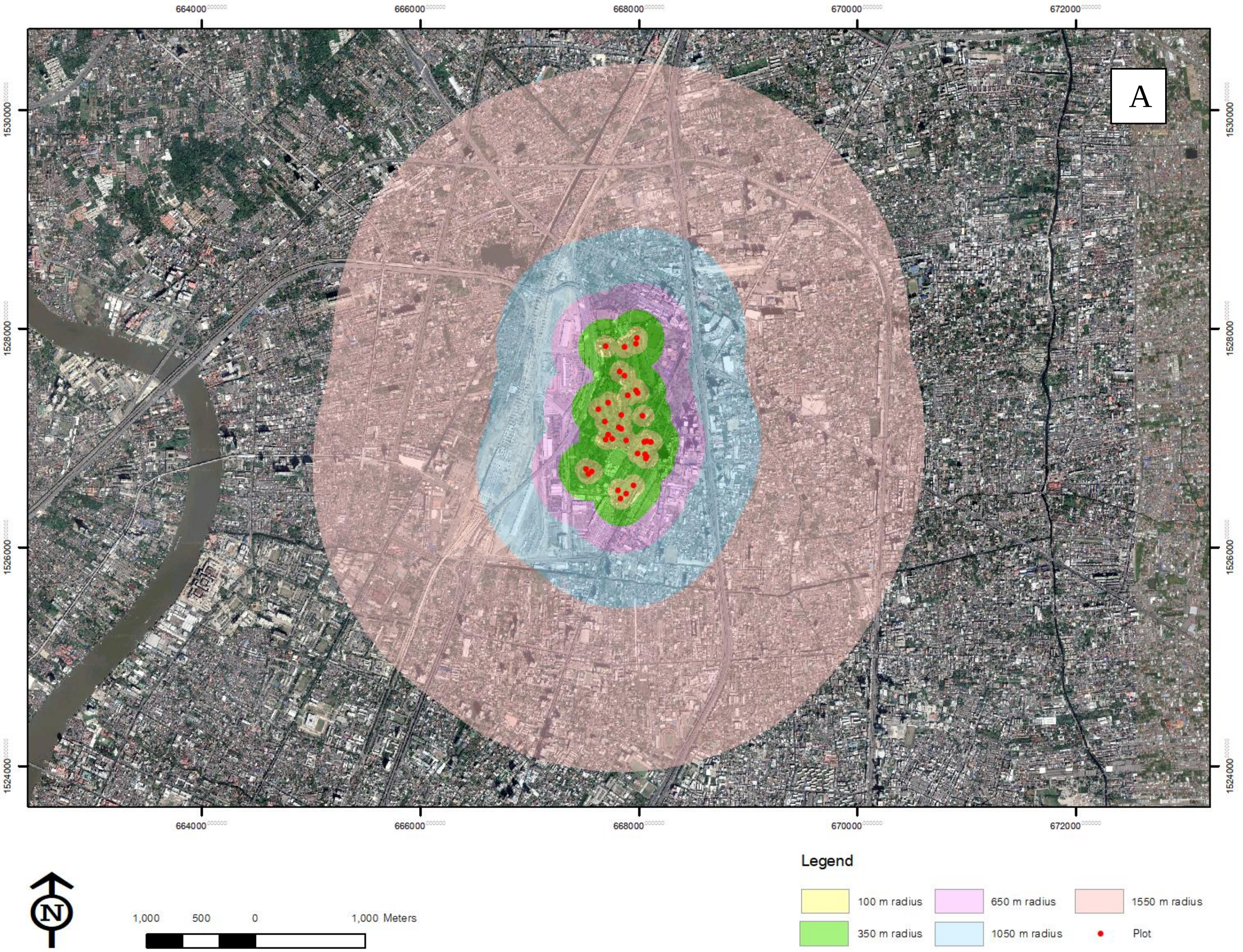

A
664000
666000
668000
670000
672000
1530000
1528000
1526000
1524000
1,000 500 0 1,000 Meters
N
Legend
100 m radius
350 m radius
650 m radius
1050 m radius
1550 m radius
Plot

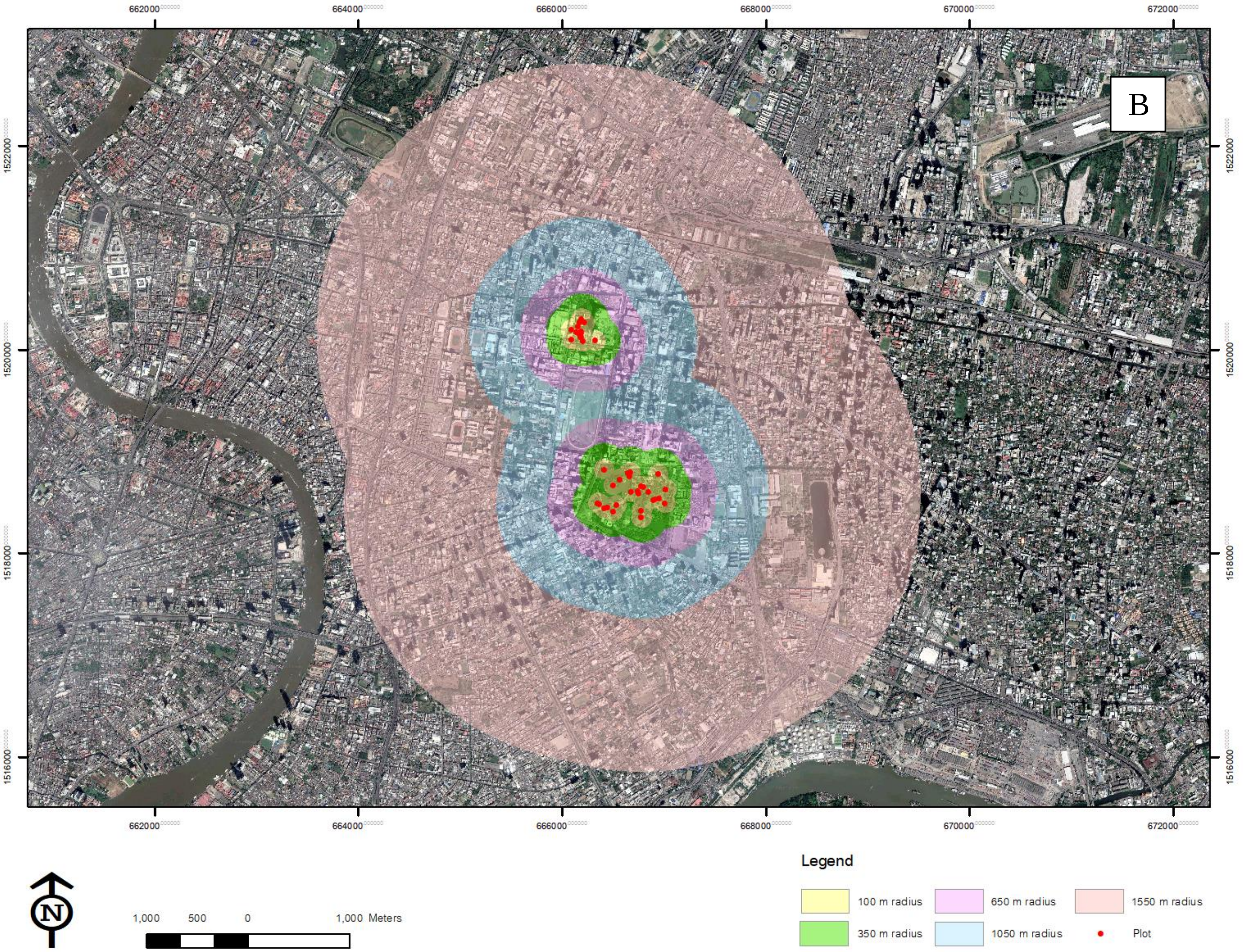
B
662000
664000
666000
668000
670000
672000
1522000
1520000
1518000
1516000
N
1,000 500 0 1,000 Meters
Legend
100 m radius
650 m radius
1550 m radius
350 m radius
1050 m radius
Plot

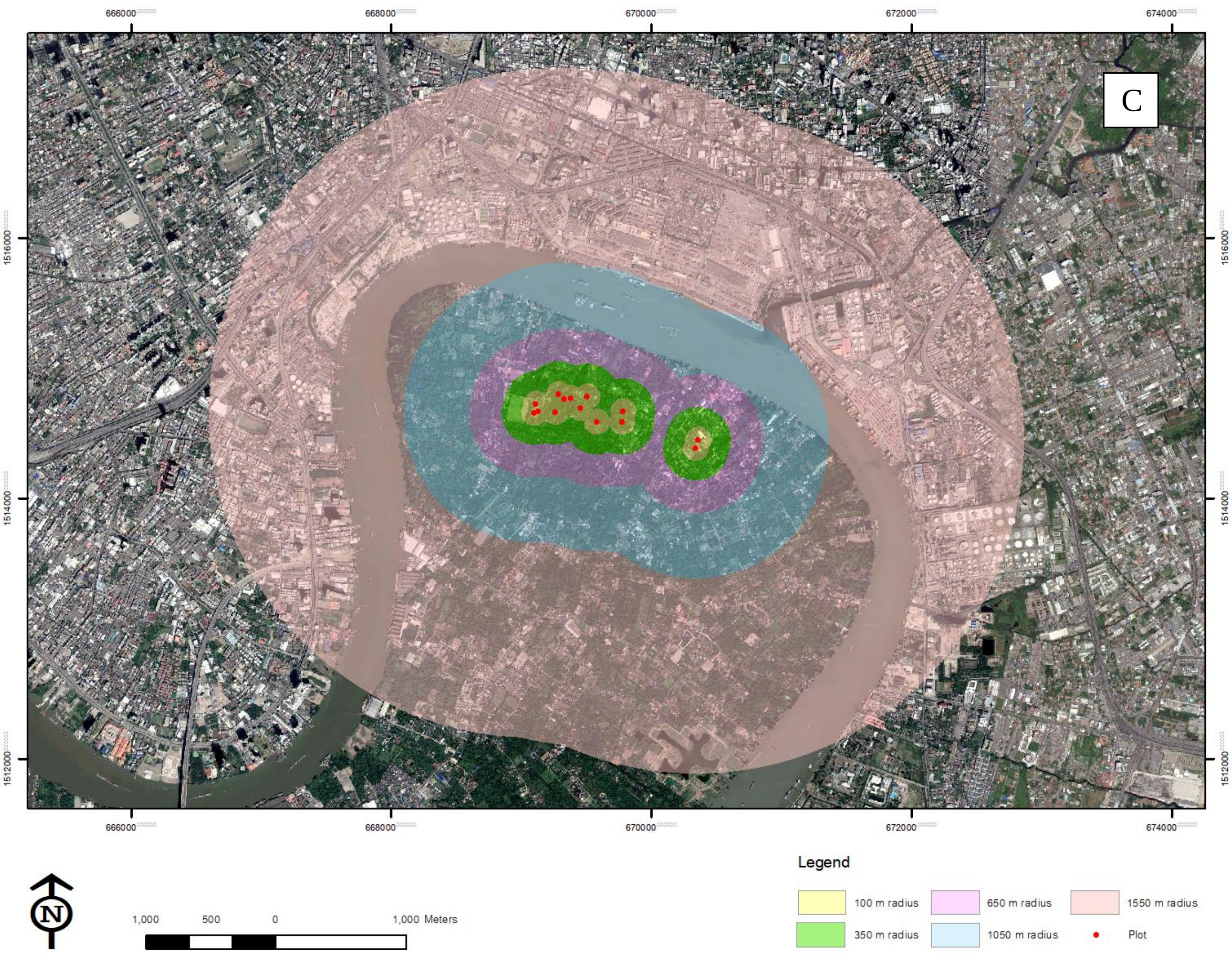

666000
668000
670000
672000
674000
1516000
1514000
1512000
C
Legend
100 m radius
650 m radius
1550 m radius
350 m radius
1050 m radius
Plot
1,000
500
0
1,000 Meters
N

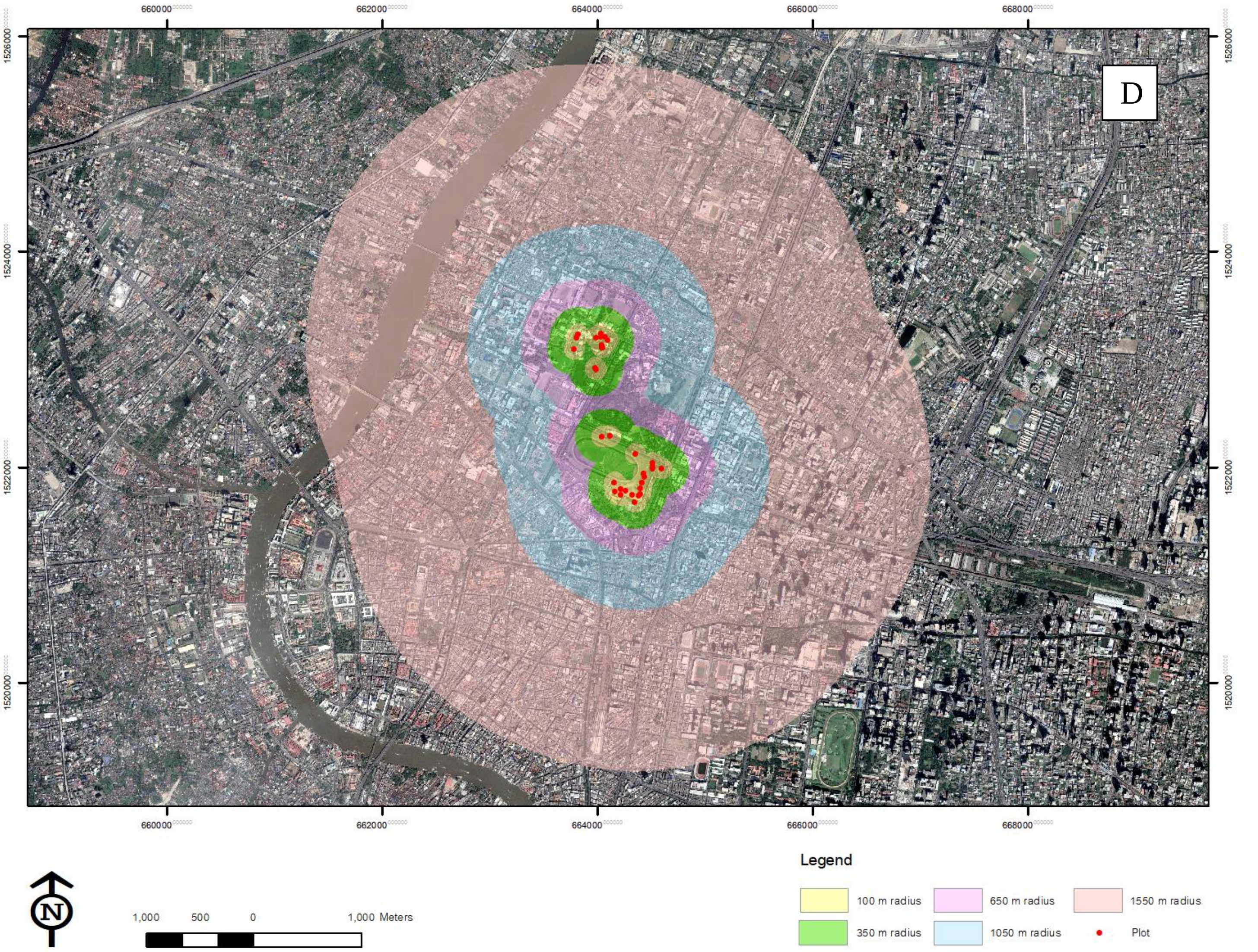

Supplemental Figure S1b. Representative samples of how we measured percent vegetation surrounding each plot within 5 concentric circles (yellow, 100 m; green, 350 m; purple, 650 m; blue, 1,050 m; and pink 1,550 m radiuses). Top to bottom: (A) Suan Wachirabenchatat, (B) Wat Pathum Wanaram and Suan Lumpini, (C) Suan Sri Nakhon Khuean Khan (left) and School Sumran Wittaya (right), and (D) Dusit Zoo and Dusit Golf Course.
