# Supplementary material for: Habitat and landscape factors influence pollinators in a tropical megacity, Bangkok, Thailand
Source: PeerJ. 2018 Jul 20;6:e5335. doi: 10.7717/peerj.5335 (PMC6055598; doi:10.7717/peerj.5335)
Supplement: Supplemental Information 2 — Supplemental Figure S2a. Two main factors (patch size and floral abundance) had significant positive effects on the abundance (number of individuals per hour) of all focal pollinator groups, except patch size did not influence stingless bee abundance. One factor (surrounding percent vegetation) generally had a significant negative effect, except it had slight positive effect on butterfly abundance. Surrounding percent vegetation best described stingless bee abundance at a 100 m radius, honey bee abundance at a 650 m radius, and both carpenter bee and butterfly abundances at a 350 m radius. Each circle represents the mean of one green area (values averaged across all plots within the green area). Supplemental Figure S2b. One additional factor, location type, influenced butterfly abundance (number of butterflies per hour), but not the abundances of any other focal taxa. Butterflies were more abundant in public parks than in commercial areas, school campuses, and temple grounds. Supplemental Figure S2c. The abundance (number of individuals per hour) of all four focal pollinator groups were influenced by a significant interaction between floral abundance and patch size. Each circle represents the mean of one green area (values averaged across all plots within the green area). The different colors and line types represent the different patch size classes: light grey (solid line) = small green areas (600–900 m2; n = 13), medium grey (dashed) = medium size green areas (901–30,000 m2; n = 13), dark grey (dot-dashed) = large green areas (30,001–81,000 m2; n = 13), black (dotted) = very large green areas (81,001–1,140,000 m2; n = 13). [file peerj-06-5335-s002.pdf]

Habitat and landscape factors influence pollinators in a tropical megacity, Bangkok, Thailand

Supplemental Figure S2. Graphs showing the significant main effects and significant interactions for each of the four focal pollinator groups (*Tetragonula* stingless bees, *Apis* honey bees, *Xylocopa* carpenter bees, and butterflies) examined in this study.

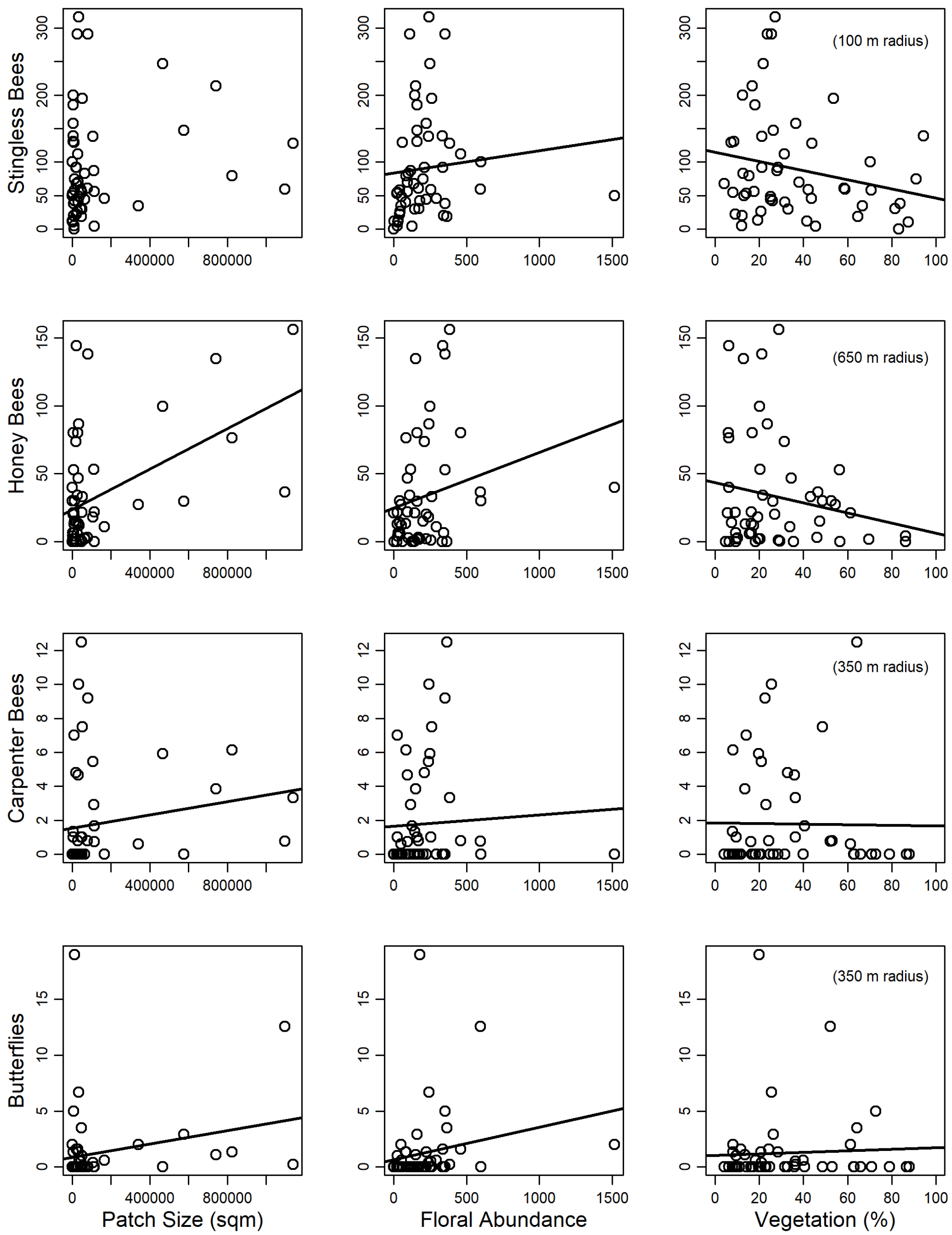

Supplemental Figure S2a. Two main factors (patch size and floral abundance) had significant positive effects on the abundance (number of individuals per hour) of all focal pollinator groups, except patch size did not influence stingless bee abundance. One factor (surrounding percent vegetation) generally had a significant negative effect, except it had slight positive effect on butterfly abundance. Surrounding percent vegetation best described stingless bee abundance at a 100 m radius, honey bee abundance at a 650 m radius, and both carpenter bee and butterfly abundances at a 350 m radius. Each circle represents the mean of one green area (values averaged across all plots within the green area).

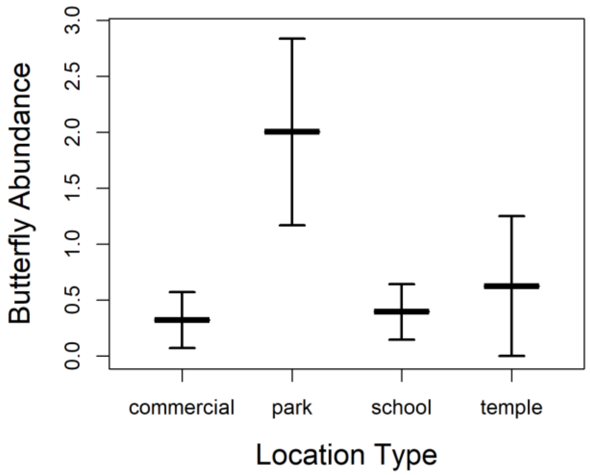

Supplemental Figure S2b. One additional factor, location type, influenced butterfly abundance (number of butterflies per hour), but not the abundances of any other focal taxa. Butterflies were more abundant in public parks than in commercial areas, school campuses, and temple grounds.

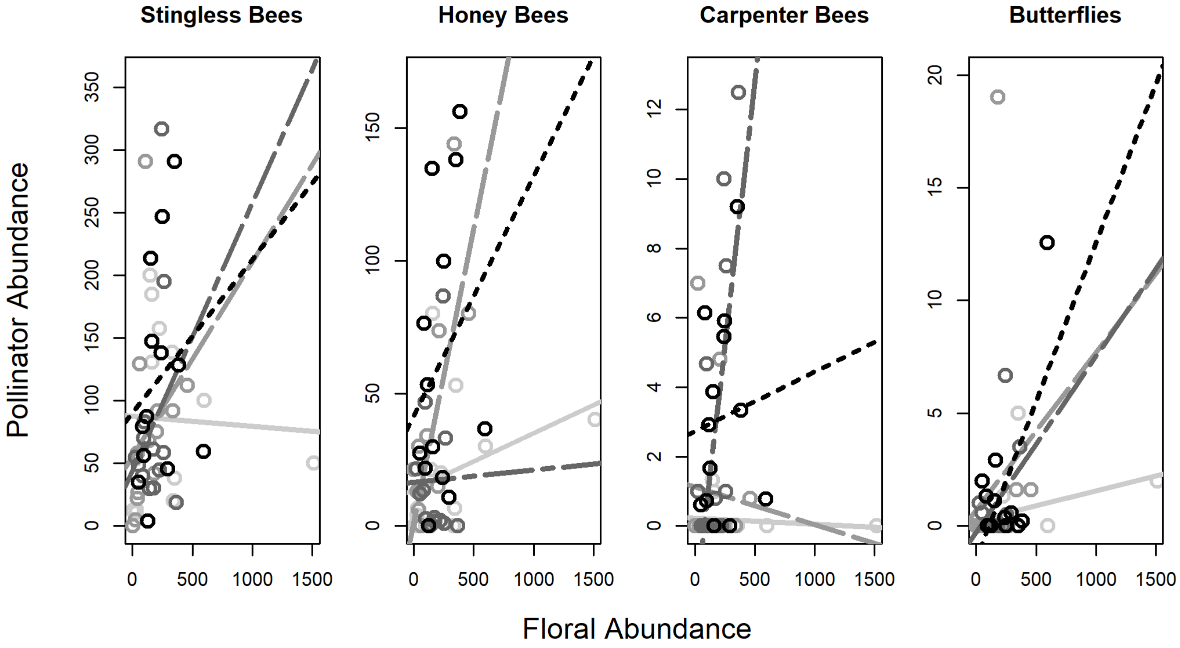

Supplemental Figure S2c. The abundance (number of individuals per hour) of all four focal pollinator groups were influenced by a significant interaction between floral abundance and patch size. Each circle represents the mean of one green area (values averaged across all plots within the green area). The different colors and line types represent the different patch size classes: light grey (solid line) = small green areas (600-900 m^2; n=13), medium grey (dashed) = medium size green areas (901-30,000 m^2; n=13), dark grey (dot-dashed) = large green areas (30,001-81,000 m^2; n=13), black (dotted) = very large green areas (81,001-1,140,000 m^2; n=13).
