# Supplementary figures and images for: Habitat and landscape factors influence pollinators in a tropical megacity, Bangkok, Thailand
Source: PeerJ. 2018 Jul 20;6:e5335. doi: 10.7717/peerj.5335 (PMC6055598; doi:10.7717/peerj.5335)

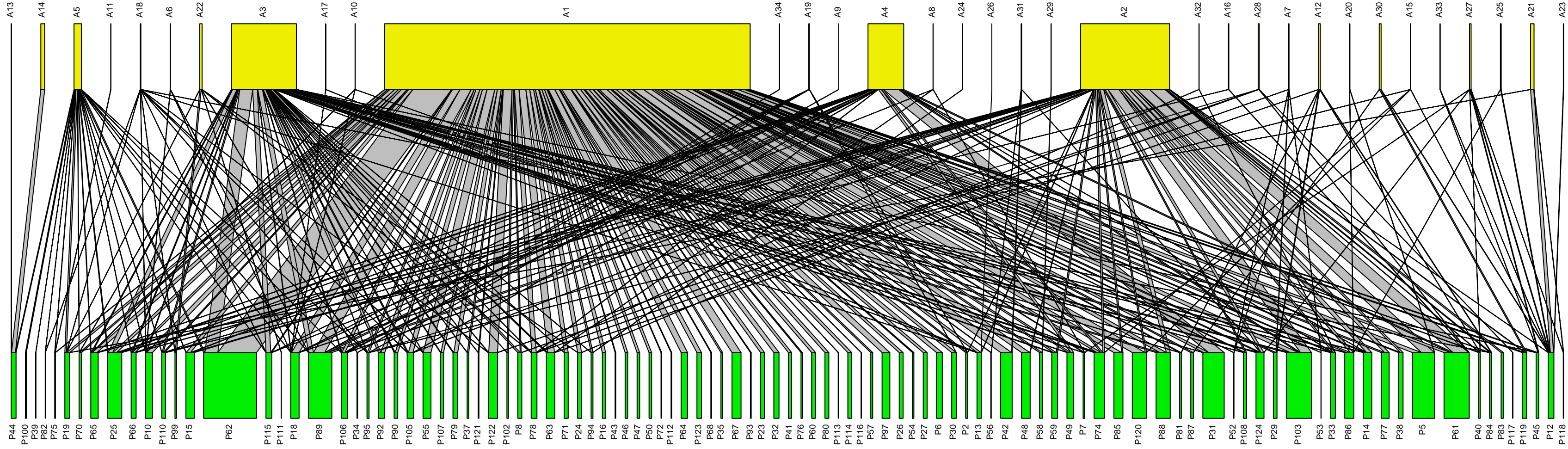

Supplement: Supplemental Information 3 — Pollinator taxa are listed along the top (yellow), and plant taxa are listed along the bottom (green). The network was constructed from pollinator abundance data at each plant species; a line connecting pollinator species i to plant species j represents an observed interaction between the two, and the width of the connecting line is proportional to the average number of i pollinator individuals observed visiting plant species j. [All species names are listed in Table S4.]. [file peerj-06-5335-s003.pdf]
