# Supplementary material for: Habitat and landscape factors influence pollinators in a tropical megacity, Bangkok, Thailand
Source: PeerJ. 2018 Jul 20;6:e5335. doi: 10.7717/peerj.5335 (PMC6055598; doi:10.7717/peerj.5335)
Supplement: Supplemental Information 4 — Green areas were categorized as either public parks, schools, temples, or commercial areas. Patch size refers to land area only (the areas of large bodies of water were subtracted from the total area). The number of plots conducted per green area was approximately proportional to the size of the green area. Floral abundance refers to the average number of flowers per plot. Pollinator richness and abundance indicate the number of pollinator species and pollinator individuals, respectively, averaged across all plots within each green area. [file peerj-06-5335-s004.pdf]

Habitat and landscape factors influence pollinators in a tropical megacity, Bangkok, Thailand

Supplemental Table S1. Descriptive information about the 52 green areas used in this study. Green areas were categorized as either public parks, schools, temples, or commercial areas. Patch size refers to land area only (the areas of large bodies of water were subtracted from the total area). The number of plots conducted per green area was approximately proportional to the size of the green area. Floral abundance refers to the average number of flowers per plot. Pollinator richness and abundance indicate the number of pollinator species and pollinator individuals, respectively, averaged across all plots within each green area.

	Green Area	Patch Size (m^2)	# of Plots	Floral Abundance	Pollinator Richness	Pollinator Abundance
**Public Parks**	Suan Wachirabenchatat	1,097,600	36	594.36	1.72	0.23
	Suan Luang R.9	741,000	29	153.66	2.38	1.57
	Suan Lumpini	576,000	26	161.31	1.50	1.90
	Suan Sri Nakhon Khuean Khan	115,000	12	126.25	0.50	1.30
	Suan Benjakitti 1	108,000	11	241.55	1.82	0.69
	Suan Rama 8	80,400	10	172.00	1.00	0.07
	Suan Benjakitti 2	66,000	8	224.25	0.88	0.30
	Public Park in Commemoration of H.M. the Kings 6th Cycle Birthday	52,000	8	25.50	1.13	0.76
	Statue King Rama 1 Park	50,000	8	145.00	0.88	0.06
	Suan Rommaninat	47,888	8	366.50	1.25	0.16
	Suan Benjasiri	46,400	8	257.63	1.13	1.25
	Suan Saranrom	36,800	7	53.43	1.29	3.22
	Suan Somdet Saranrat Maneerom	34,500	6	245.00	1.83	0.60
	Suan Santiphap	32,000	6	84.33	0.83	0.21
	Suan Chaloemphrakiat 80 Punsa Por Thek Thung	31,100	6	97.00	1.67	1.46
	Suan Chalermprakiat 80 years	27,200	6	110.50	1.67	0.90
	Suan Phaya Thai Pirom	28,600	5	460.00	1.60	0.34
	Suan Vibhavadi Rangsit Forest Park	23,300	5	140.00	0.80	0.20
	Suan Phan Phirom	22,400	5	339.00	1.80	0.28
	Suan Chaloem Phrakiat Forest Park	20,500	5	211.00	2.60	0.37
	Suan Santichai Prakan	13,600	4	181.00	1.50	0.29
	Suan Princess Mothers Commemoration Public Park	12,900	4	42.50	1.75	0.54

	Green Area	Patch Size (m^2)	# of Plots	Floral Abundance	Pollinator Richness	Pollinator Abundance
	Suan Pakkard Palace	9,600	4	26.50	1.25	0.30
	Suan Serene Garden Mithra	4,864	4	161.00	1.50	1.50
	Princess Mother Memorial Park	7,920	3	343.33	1.67	0.08
	Suan Charanphirom	4,800	3	225.00	2.67	0.50
	Suan Sra Keaw Community	968	2	600.00	1.00	0.03
**Schools**	Kasetsart University	1,140,000	36	385.17	2.50	0.67
	Chulalongkorn University	824,000	30	84.90	2.37	0.72
	Ramkhamhaeng University	467,000	23	250.83	2.04	0.93
	Thai Japanese Bangkok Youth Center	114,000	11	96.36	1.64	0.30
	Srinakharinwirot University	112,000	11	116.82	1.73	0.78
	Thammasat University Tha Prachan	81,300	10	352.80	2.70	1.79
	Mahidol University	64,700	9	101.56	1.00	0.30
	School Sumran Wittaya	1,000	2	1515.00	2.00	0.67
	Thai Women Association School	600	1	4.00	1.00	0.75
**Temples**	Wat Pho	54,000	8	264.00	2.25	0.34
	Wat Pathum Wanaram	26,700	8	44.38	0.75	0.20
	Wat Arun	21,400	8	41.25	1.25	2.95
	Wat Thepsirin	43,900	7	175.71	0.86	0.10
	Wat Saket	12,600	4	202.50	0.75	0.98
	Wat Suthat	8,600	4	353.25	1.75	1.76
	Wat Rakang	10,900	3	0.67	0.33	2.67
	Wat Thepthidaram	6,700	3	334.00	0.67	0.70
**Commercial Areas**	Dusit Golf Course	343,000	20	51.65	1.05	0.47
	Dusit Zoo	167,000	14	293.21	1.29	0.30
	Par 3 Srinakarin Golf	10,000	3	61.67	1.33	0.54
	Royal Pavilion Mahajetsadabadin	6,020	3	161.33	2.33	0.50
	Royal Railway Station	5,472	3	145.67	2.67	1.03
	Lan Pra Ratcha Nu Saowaree	4,700	3	34.00	0.67	1.68
	Siriraj Hospital	4,470	3	25.00	0.67	0.47
	Wong Wian 22	4,430	3	30.00	1.33	0.24
