# Supplementary material for: Habitat and landscape factors influence pollinators in a tropical megacity, Bangkok, Thailand
Source: PeerJ. 2018 Jul 20;6:e5335. doi: 10.7717/peerj.5335 (PMC6055598; doi:10.7717/peerj.5335)
Supplement: Supplemental Information 5 — Taxa are sorted alphabetically by family and then species name. Frequency refers to the number of 2x2 m plots in which each plant taxa was encountered. Pollinator richness and pollinator abundance indicate the average number of pollinator species and pollinator individuals, respectively, recorded at each plant taxa during the 15-minute observation period. We do no have pollinator richness and abundance values for some plant taxa (“NA”) because they only occurred in plots with other plant species, and we did not separate the pollinator data by plant species in multi-species plots. Therefore, we only have pollinator richness and abundance values for plant taxa that were observed in a single-species plot at least once. Distribution information was obtained from the Catalogue of Life website (http://www.catalogueoflife.org; accessed May 2018): native–listed as native in Thailand or Southeast Asia; exotic–listed as introduced in Thailand or Southeast Asia; unclear–distribution data is not clear (e.g., listed as native in certain neighboring countries, but no information reported for Thailand); no info–no distribution information listed at all. For the columns with numeric information, the five highest values are highlighted in yellow (Frequency), blue (Pollinator Richness), and pink (Pollinator Abundance). [file peerj-06-5335-s005.pdf]

Habitat and landscape factors influence pollinators in a tropical megacity, Bangkok, Thailand

Supplemental Table S2. Detailed information about the 140 plant taxa observed in this study. Taxa are sorted alphabetically by family and then species name. Frequency refers to the number of 2x2 m plots in which each plant taxa was encountered. Pollinator richness and pollinator abundance indicate the average number of pollinator species and pollinator individuals, respectively, recorded at each plant taxa during the 15-minute observation period. We do no have pollinator richness and abundance values for some plant taxa ("NA") because they only occurred in plots with other plant species, and we did not separate the pollinator data by plant species in multi-species plots. Therefore, we only have pollinator richness and abundance values for plant taxa that were observed in a single-species plot at least once. Distribution information was obtained from the Catalogue of Life website (http://www.catalogueoflife.org; accessed May 2018): native – listed as native in Thailand or Southeast Asia; exotic – listed as introduced in Thailand or Southeast Asia; unclear – distribution data is not clear (e.g., listed as native in certain neighboring countries, but no information reported for Thailand); no info – no distribution information listed at all. For the columns with numeric information, the five highest values are highlighted in yellow (Frequency), blue (Pollinator Richness), and pink (Pollinator Abundance).

Family	Plant Species	Frequency (# of plots)	Pollinator Richness	Pollinator Abundance	Distribution
Acanthaceae	*Asystasia gangetica* (L.) T.Anderson	8	1.50	0.27	native
	Crossandra nilotica Oliv.	1	0.00	0.00	exotic
	Graptophyllum pictum (L.) Griff.	1	1.00	0.25	exotic
	Hygrophila erecta (Burm.f.) Hochr.	4	1.25	0.26	exotic
	Justicia betonica L.	1	5.00	0.13	exotic
	Pseuderanthemum curtatum (C. B. Cl.) Merrill	3	0.67	0.08	unclear
	Ruellia simplex C.Wright	46	1.32	0.38	exotic
	Thunbergia grandiflora (Roxb. ex Rottl.) Roxb.	1	1.00	1.00	unclear
	Thunbergia laurifolia Lindl.	1	1.00	1.20	unclear
Alismataceae	*Echinodorus cordifolius* (L.) Griseb.	6	2.00	0.47	exotic
Amaranthaceae	*Amaranthus viridis* L.	1	3.00	0.09	exotic
	Gomphrena celosioides Mart.	4	1.67	0.23	exotic
	Gomphrena globosa L.	1	1.00	0.57	native
Amaryllidaceae	*Crinum asiaticum* L.	6	0.80	2.62	native

Family	Plant Species	Frequency (# of plots)	Pollinator Richness	Pollinator Abundance	Distribution
	Hymenocallis littoralis (Jacq.) Salisb.	2	1.00	0.14	no info
	Zephyranthes minuta (Kunth) D.Dietr.	1	1.00	1.40	exotic
Anacardiaceae	*Mangifera indica* L.	1	1.00	0.04	native
Apocynaceae	*Adenium obesum* (Forssk.) Roem. & Schult.	1	0.00	0.00	exotic
	Allamanda cathartica L.	3	0.00	0.00	exotic
	Cascabela thevetia (L.) Lippold	1	0.00	0.00	exotic
	Catharanthus roseus (L.) G.Don	2	0.00	0.00	exotic
	Cerbera odollam Gaertn.	1	0.00	0.00	native
	Marsdenia floribunda (Brongn.) Schltr.	1	0.00	0.00	exotic
	Nerium oleander L.	7	0.29	0.02	exotic
	Plumeria spp.	8	0.00	0.00	exotic
	Tabernaemontana pandacaqui Lam.	2	NA	NA	native
	Vallaris glabra (L.) Kuntze	1	NA	NA	unclear
	Wrightia religiosa (Teijsm. & Binn.) Benth. ex Kurz	19	0.72	0.17	unclear
Bignoniaceae	*Handroanthus chrysanthus* (Jacq.) S.O.Grose	2	2.00	0.66	exotic
	Jacaranda sp.	1	NA	NA	exotic
	Podranea ricasoliana (Tanfani) Sprague	1	NA	NA	exotic
	Spathodea campanulata P.Beauv.	2	1.50	0.43	exotic
	Tabebuia aurea (Silva Manso) Benth. & Hook.f. ex S.Moore	8	1.63	0.40	exotic
	Tabebuia rosea (Bertol.) Bertero ex A.DC.	16	2.53	0.83	exotic
	Tecoma stans (L.) Juss. ex Kunth	8	2.00	0.39	exotic
Boraginaceae	*Cordia alba* (Jacq.) Roem. & Schult.	1	3.00	0.83	exotic
	Cordia sebestena L.	1	1.00	0.29	exotic
	Ehretia microphylla Lam.	1	1.00	0.63	native
Cannaceae	*Canna indica* L.	2	1.00	3.46	exotic
Capparaceae	*Crateva religiosa* G.Forst.	1	2.00	0.01	native
Cleomaceae	*Cleome spinosa* Jacq.	1	1.00	0.04	exotic
Clusiaceae	*Calophyllum inophyllum* L.	3	3.50	1.40	native

Family	Plant Species	Frequency (# of plots)	Pollinator Richness	Pollinator Abundance	Distribution
Combretaceae	*Combretum indicum* (L.) DeFilipps	3	1.00	0.03	native
Compositae	*Cosmos sulphureus* Cav.	1	NA	NA	exotic
	Cyanthillium cinereum (L.) H.Rob.	1	NA	NA	native
	Emilia sonchifolia (L.) DC. ex DC.	1	NA	NA	native
	Helianthus annuus L.	1	1.00	2.50	exotic
	Pluchea indica (L.) Less.	1	2.00	0.08	native
	Tagetes erecta L.	4	2.00	0.93	exotic
	Tridax procumbens (L.) L.	3	NA	NA	exotic
Convolvulaceae	*Evolvulus nummularius* (L.) L.	1	NA	NA	exotic
	Ipomoea aquatica Forssk.	5	0.75	2.37	native
	Ipomoea carnea Jacq.	2	1.50	0.88	no info
Costaceae	*Cheilocostus speciosus* (J.Koenig) C.D.Specht	1	1.00	0.50	native
Cucurbitaceae	*Coccinia grandis* (L.) Voigt	4	2.50	2.94	native
	Luffa cylindrica (L.) M.Roem.	1	3.00	4.50	exotic
Euphorbiaceae	*Jatropha integerrima* Jacq.	4	2.00	1.55	exotic
	Jatropha multifida L.	1	1.00	0.12	exotic
Lamiaceae	*Clerodendrum* x *speciosum*	1	1.00	0.09	exotic
	Ocimum × *africanum* Lour.	2	2.00	0.23	native
	Ocimum basilicum L.	5	3.00	0.50	native
	Ocimum tenuiflorum L.	2	2.00	0.03	native
	Salvia farinacea Benth.	1	1.00	0.30	exotic
	Vitex negundo L.	1	3.00	0.20	native
Lecythidaceae	*Couroupita guianensis* Aubl.	7	2.14	1.76	exotic
	Gustavia gracillima Miers	2	1.50	22.17	exotic
Leguminosae	*Albizia saman* (Jacq.) Merr.	11	1.00	0.14	no info
	Bauhinia acuminata L.	1	0.00	0.00	exotic
	Bauhinia purpurea L.	5	2.00	0.75	exotic
	Caesalpinia pulcherrima (L.) Sw.	28	1.35	0.44	no info
	Cassia fistula L.	32	1.76	0.28	no info

Family	Plant Species	Frequency (# of plots)	Pollinator Richness	Pollinator Abundance	Distribution
	Cassia surattensis Burm.f.	5	1.80	0.20	no info
	Clitoria ternatea L.	1	0.00	0.00	no info
	Delonix regia (Hook.) Raf.	7	1.11	0.24	exotic
	Erythrina variegata L.	1	2.00	1.27	no info
	Gliricidia sepium (Jacq.) Walp.	1	3.00	0.03	no info
	Leucaena leucocephala (Lam.) de Wit	2	0.00	0.00	no info
	Parkinsonia aculeata L.	1	3.00	2.75	no info
	Peltophorum pterocarpum (DC.) K.Heyne	19	4.17	0.56	no info
	Pterocarpus indicus Willd.	5	1.40	0.02	no info
	Saraca asoca (Roxb.) Willd.	2	0.00	0.00	no info
	Saraca indica L.	2	1.50	0.03	no info
	Senna siamea (Lam.) H.S.Irwin & Barneby	3	1.00	0.09	no info
	Sesbania grandiflora (L.) Pers.	1	0.00	0.00	no info
	Tamarindus indica L.	1	3.00	0.83	no info
Linderniaceae	*Torenia fournieri* Linden ex E. Fourn.	1	NA	NA	native
Lythraceae	*Lagerstroemia calyculata* Kurz	1	3.00	0.21	native
	Lagerstroemia indica L.	6	1.60	0.39	native
	Lagerstroemia sp.	1	1.00	0.33	native
	Lagerstroemia speciosa (L.) Pers.	17	2.31	0.69	no info
	Lagerstroemia tomentosa C. Presl	3	2.67	1.26	native
	Lagerstroemia hybrid	1	1.00	0.35	native
Malpighiaceae	*Galphimia glauca* Cav.	1	1.00	0.00	exotic
	Malpighia coccigera L.	1	2.00	0.10	exotic
Malvaceae	*Alcea rosea* L.	1	0.00	0.00	exotic
	Dombeya elegans Cordem.	1	2.00	0.12	no info
	Hibiscus rosa-sinensis L.	5	0.00	0.00	exotic
	Thespesia populnea (L.) Sol. ex Corrêa	1	1.00	0.83	native
Marantaceae	*Thalia geniculata* L.	1	1.00	0.03	exotic
Moringaceae	*Moringa oleifera* Lam.	2	0.50	0.20	exotic

Family	Plant Species	Frequency (# of plots)	Pollinator Richness	Pollinator Abundance	Distribution
Muntingiaceae	*Muntingia calabura* L.	3	2.00	1.86	exotic
Musaceae	*Musa paradisiaca* L.	3	1.33	1.61	native
	Musa rubra Wall. ex Kurz	1	1.00	1.50	native
Myrtaceae	*Callistemon viminalis* (Sol. ex Gaertn.) G.Don	2	3.00	0.37	exotic
	Syzygium jambos (L.) Alston	2	4.00	2.93	exotic
	Syzygium malaccense (L.) Merr. & L.M.Perry	1	0.00	0.00	native
	Xanthostemon chrysanthus (F.Muell.) Benth.	5	1.80	0.39	exotic
Nelumbonaceae	*Nelumbo nucifera* Gaertn.	4	2.00	9.75	exotic
Nyctaginaceae	*Bougainvillea* spp.	6	0.00	0.00	exotic
Nymphaeaceae	*Nymphaea lotus* L.	3	1.00	6.14	native
	Nymphaea nouchali Burm.f.	7	2.00	10.10	native
Oxalidaceae	*Averrhoa carambola* L.	1	3.00	0.03	exotic
Passifloraceae	*Turnera ulmifolia* L.	5	2.33	0.53	exotic
Plantaginaceae	*Angelonia goyazensis* Benth.	2	1.00	0.11	exotic
	Russelia equisetiformis Schltdl. & Cham.	2	0.00	0.00	exotic
Poaceae	*Oryza* sp.	1	NA	NA	-
	Poaceae spp.	2	NA	NA	-
	Zoysia japonica Steud.	7	2.33	0.13	exotic
Portulacaceae	*Portulaca oleracea* L.	4	2.00	0.59	native
Rosaceae	*Rosa* sp.	1	NA	NA	-
Rubiaceae	*Arachnothryx leucophylla* (Kunth) Planch.	1	NA	NA	exotic
	Ixora coccinea L.	6	0.67	0.06	native
	Ixora finlaysoniana Wall. ex G.Don	2	1.00	0.45	no info
	Ixora sp.	1	2.00	0.04	-
	Morinda citrifolia L.	1	3.00	0.24	native
	Tamilnadia uliginosa (Retz.) Tirveng. & Sastre	1	0.00	0.00	native
Rutaceae	*Citrus maxima* (Burm.) Merr.	1	5.00	3.67	exotic
	Murraya paniculata (L.) Jack	6	1.83	0.52	native
Sapindaceae	*Lepisanthes fruticosa* (Roxb.) Leenh.	1	NA	NA	native

Family	Plant Species	Frequency (# of plots)	Pollinator Richness	Pollinator Abundance	Distribution
Sapotaceae	*Mimusops elengi* L.	5	3.00	0.84	native
Scrophulariaceae	*Buddleja paniculata* Wall.	2	4.50	0.01	native
Verbenaceae	*Citharexylum spinosum* L.	1	3.00	0.03	exotic
	Duranta erecta L.	2	0.50	0.00	exotic
	Lantana camara L.	3	1.00	0.02	exotic
Vitaceae	*Cayratia trifolia* (L.) Domin	1	0.00	0.00	native
-	Unknown 1	1	1.00	0.25	-
-	Unknown 6	1	3.00	0.58	-
-	Unknown 11	1	1.00	0.00	-
-	Unknown 13	1	2.00	0.00	-
-	Unknown 15	1	NA	NA	-
