# Supplementary material for: Habitat and landscape factors influence pollinators in a tropical megacity, Bangkok, Thailand
Source: PeerJ. 2018 Jul 20;6:e5335. doi: 10.7717/peerj.5335 (PMC6055598; doi:10.7717/peerj.5335)
Supplement: Supplemental Information 6 — Taxa are sorted numerically by “Order” (highest to lowest percentage), then alphabetically by “Family,” then numerically by “Frequency.” The frequencies of some taxa are unknown, as they were only identified to genus or family in the field, and were later identified to species or genus in the lab based on collected or photographed specimens. The nine most common taxa are highlighted in yellow. Distribution information was obtained from the Catalogue of Life website (http://www.catalogueoflife.org; accessed May 2018). All pollinator taxa that could be sufficiently identified are native to Thailand. [file peerj-06-5335-s006.pdf]

Habitat and landscape factors influence pollinators in a tropical megacity, Bangkok, Thailand

Supplemental Table S3. Detailed information about each of the pollinator taxa observed in this study. Taxa are sorted numerically by "Order" (highest to lowest percentage), then alphabetically by "Family", then numerically by "Frequency". The frequencies of some taxa are unknown, as they were only identified to genus or family in the field, and were later identified to species or genus in the lab based on collected or photographed specimens. The nine most common taxa are highlighted in yellow. Distribution information was obtained from the Catalogue of Life website (http://www.catalogueoflife.org; accessed May 2018). All pollinator taxa that could be sufficiently identified are native to Thailand.

Order	Family	Genus	Frequency (# of individuals)	Percent	Distribution
Hymenoptera (98.6%)	Apidae	*Tetragonula* spp.	12,087	64.317	native
		Apis florea Fabricius, 1787	2,441	12.989	native
		Apis cerana Fabricius, 1793	2,345	12.478	native
		Apis dorsata Fabricius, 1793	1,148	6.109	native
		Xylocopa aestuans Linnaeus, 1758	273	1.453	native
		Xylocopa nasalis Westwood, 1842	12	0.064	native
		Ceratina spp.	9	0.048	-
		Ceratina cognata Smith, 1879	(specimen)	-	native
		Ceratina dentipes Friese, 1914	(specimen)	-	native
		Amegilla spp.	7	0.037	-
	Crabronidae	spp.	2	0.011	-
	Eumenidae	*Delta conoideum* Gmelin, 1790	32	0.170	native
		Delta pyriforme Fabricius, 1775	2	0.011	native
		Polistes spp.	2	0.011	-
	Halictidae	*Lasioglossum* spp.	84	0.447	-
		Nomia spp.	3	0.016	-
	Megachilidae	*Megachile* spp.	30	0.160	-
	Scoliidae	*Campsomeris* spp.	11	0.059	-
		spp.	6	0.032	-

	Unknown	spp.	27	0.144	-
	Vespidae	spp.	15	0.080	-
		Ropalidia spp.	(specimen)	-	-
Lepidoptera (1.16%)	Hesperiidae	spp.	1	0.005	-
	Lycaenidae	spp.	57	0.303	-
		Chilades pandava Horsfield, 1829	14	0.074	native
	Nymphalidae	*Danaus chrysippus* Linnaeus, 1758	54	0.287	native
		Junonia lemonias Linnaeus, 1758	14	0.074	native
		Ideopsis similis Linnaeus, 1758	2	0.011	native
	Papilionidae	*Graphium doson* (Felder & Felder, 1864)	5	0.027	native
	Pieridae	*Delias eucharis* Drury, 1773	43	0.229	native
		Catopsilia Pomona Fabricius, 1775	17	0.090	native
	Unknown	spp.	11	0.059	-
Diptera (0.15%)	Sacrophagidae	spp.	2	0.011	-
	Syrphidae	spp.	20	0.106	-
		Eristalinus arvorum Fabricius, 1787	(specimen)	-	native
	Unknown	spp.	6	0.032	-
Coleoptera (0.032%)	Chrysomelidae	sp.	1	0.005	-
	Curculionoidea	spp.	5	0.027	-
Hemiptera (0.021%)	Lygaeidae	spp.	4	0.021	-
Passeriformes (0.005%)	Nectariniidae	*Cinnyris jugularis* Linnaeus, 1766	1	0.005	native
