# Supplementary material for: Habitat and landscape factors influence pollinators in a tropical megacity, Bangkok, Thailand
Source: PeerJ. 2018 Jul 20;6:e5335. doi: 10.7717/peerj.5335 (PMC6055598; doi:10.7717/peerj.5335)
Supplement: Supplemental Information 7 — Table S4a. Details and results of the pollinator taxa used in the pollination network. “Label” refers to the labels used for the pollinator taxa in the pollination network (Fig. 3; Fig. S3). Degree refers to the number of floral hosts per pollinator taxa. Normalized degree is calculated as degree divided by the number of possible interacting partners, which accounts for differences in network size. Table S4b. Details of the plant taxa used in the pollination network. “Label” refers to the labels used for the plant taxa in the pollination network (Fig. 3; Fig. S3). [file peerj-06-5335-s007.pdf]

Habitat and landscape factors influence pollinators in a tropical megacity, Bangkok, Thailand

Supplemental Table S4. Details and results of the pollination network analysis.

Table S4a. Details and results of the pollinator taxa used in the pollination network. "Label" refers to the labels used for the pollinator taxa in the pollination network (Figure 3, S3). Degree refers to the number of floral hosts per pollinator taxa. Normalized degree is calculated as degree divided by the number of possible interacting partners, which accounts for differences in network size.

Label	Pollinator taxa	Degree	Normalized Degree
A1	*Tetragonula*	93	0.894231
A2	*Apis florea*	33	0.317308
A3	*Apis cerana*	42	0.403846
A4	*Apis dorsata*	17	0.163462
A5	*Xylocopa aestuans*	19	0.182692
A6	*Xylocopa nasalis*	3	0.028846
A7	*Ceratina*	4	0.038462
A8	*Amegilla*	3	0.028846
A9	Crabronidae	1	0.009615
A10	*Delta*	3	0.028846
A11	*Polistes*	1	0.009615
A12	*Lasioglossum*	6	0.057692
A13	*Nomia*	1	0.009615
A14	*Megachile*	1	0.009615
A15	*Campsomeris*	4	0.038462
A16	Scoliidae	2	0.019231
A17	*Ropalidia*	2	0.019231
A18	Unknown Hymenoptera	11	0.105769
A19	*Catopsilia*	6	0.057692
A20	*Chilades*	2	0.019231
A21	*Danaus*	3	0.028846
A22	*Delias*	6	0.057692
A23	*Graphium*	1	0.009615
A24	*Ideopsis*	1	0.009615
A25	*Junonia*	2	0.019231
A26	Hesperiidae	1	0.009615
A27	Lycaenidae	6	0.057692
A28	Unknown Butterfly	5	0.048077
A29	Sacrophagidae	1	0.009615
A30	Syrphidae	3	0.028846
A31	Unknown Diptera	3	0.028846
A32	Coleoptera	2	0.019231
A33	Hemiptera	2	0.019231
A34	Sunbird	1	0.009615

Table S4b. Details of the plant taxa used in the pollination network. "Label" refers to the labels used for the plant taxa in the pollination network (Figure 3, S3).

Label	Plant Species
P1	*Adenium obesum* (Forssk.) Roem. & Schult.
P2	*Albizia saman* (Jacq.) Merr.
P3	*Alcea rosea* L.
P4	*Allamanda cathartica* L.
P5	*Amaranthus viridis* L.
P6	*Angelonia goyazensis* Benth.
P7	*Asystasia gangetica* (L.) T.Anderson
P8	*Averrhoa carambola* L.
P9	*Bauhinia acuminata* L.
P10	*Bauhinia purpurea* L.
P11	*Bougainvillea*
P12	*Buddleja paniculata* Wall.
P13	*Caesalpinia pulcherrima* (L.) Sw.
P14	*Callistemon viminalis* (Sol. ex Gaertn.) G.Don
P15	*Calophyllum inophyllum* L.
P16	*Canna indica* L.
P17	*Cascabela thevetia* (L.) Lippold
P18	*Cassia fistula* L.
P19	*Cassia surattensis* Burm.f.
P20	*Catharanthus roseus* (L.) G.Don
P21	*Cayratia trifolia* (L.) Domin
P22	*Cerbera odollam* Gaertn.
P23	*Cheilocostus speciosus* (J.Koenig) C.D.Specht
P24	*Citharexylum spinosum* L.
P25	*Citrus maxima* (Burm.) Merr.
P26	*Cleome spinosa* Jacq.
P27	*Clerodendrum* x *speciosum*
P28	*Clitoria ternatea* L.
P29	*Coccinia grandis* (L.) Voigt
P30	*Combretum indicum* (L.) DeFilipps
P31	*Cordia alba* (Jacq.) Roem. & Schult.
P32	*Cordia sebestena* L.
P33	*Couroupita guianensis* Aubl.
P34	*Crateva religiosa* G.Forst.
P35	*Crinum asiaticum* L.
P36	*Crossandra nilotica* Oliv.

Label	Plant Species
P37	*Delonix regia* (Hook.) Raf.
P38	*Dombeya elegans* Cordem.
P39	*Duranta erecta* L.
P40	*Echinodorus cordifolius* (L.) Griseb.
P41	*Ehretia microphylla* Lam.
P42	*Erythrina variegata* L.
P43	*Galphimia glauca* Cav.
P44	*Gliricidia sepium* (Jacq.) Walp.
P45	*Gomphrena celosioides* Mart.
P46	*Gomphrena globosa* L.
P47	*Graptophyllum pictum* (L.) Griff.
P48	*Gustavia gracillima* Miers
P49	*Handroanthus chrysanthus* (Jacq.) S.O.Grose
P50	*Helianthus annuus* L.
P51	*Hibiscus rosa-sinensis* L.
P52	*Hygrophila erecta* (Burm.f.) Hochr.
P53	*Hymenocallis littoralis* (Jacq.) Salisb.
P54	*Ipomoea aquatica* Forssk.
P55	*Ipomoea carnea* Jacq.
P56	*Ixora coccinea* L.
P57	*Ixora finlaysoniana* Wall. ex G.Don
P58	*Ixora* sp.
P59	*Jatropha integerrima* Jacq.
P60	*Jatropha multifida* L.
P61	*Justicia betonica* L.
P62	*Lagerstroemia calyculata* Kurz
P63	*Lagerstroemia indica* L.
P64	*Lagerstroemia* sp.
P65	*Lagerstroemia speciosa* (L.) Pers.
P66	*Lagerstroemia tomentosa* C. Presl
P67	*Lagerstroemia* hybrid
P68	*Lantana camara* L.
P69	*Leucaena leucocephala* (Lam.) de Wit
P70	*Luffa cylindrica* (L.) M.Roem.
P71	*Malpighia coccigera* L.
P72	*Mangifera indica* L.
P73	*Marsdenia floribunda* (Brongn.) Schltr.
P74	*Mimusops elengi* L.
P75	*Morinda citrifolia* L.

Label	Plant Species
P76	*Moringa oleifera* Lam.
P77	*Muntingia calabura* L.
P78	*Murraya paniculata* (L.) Jack
P79	*Musa paradisiaca* L.
P80	*Musa rubra* Wall. ex Kurz
P81	*Nelumbo nucifera* Gaertn.
P82	*Nerium oleander* L.
P83	*Nymphaea lotus* L.
P84	*Nymphaea nouchali* Burm.f.
P85	*Ocimum* × *africanum* Lour.
P86	*Ocimum basilicum* L.
P87	*Ocimum tenuiflorum* L.
P88	*Parkinsonia aculeata* L.
P89	*Peltophorum pterocarpum* (DC.) K.Heyne
P90	*Pluchea indica* (L.) Less.
P91	*Plumeria* spp.
P92	*Portulaca oleracea* L.
P93	*Pseuderanthemum curtatum* (C. B. Cl.) Merrill
P94	*Pterocarpus indicus* Willd.
P95	*Ruellia simplex* C.Wright
P96	*Russelia equisetiformis* Schltdl. & Cham.
P97	*Salvia farinacea* Benth.
P98	*Saraca asoca* (Roxb.) Willd.
P99	*Saraca indica* L.
P100	*Senna siamea* (Lam.) H.S.Irwin & Barneby
P101	*Sesbania grandiflora* (L.) Pers.
P102	*Spathodea campanulata* P.Beauv.
P103	*Syzygium jambos* (L.) Alston
P104	*Syzygium malaccense* (L.) Merr. & L.M.Perry
P105	*Tabebuia aurea* (Silva Manso) Benth. & Hook.f. ex S.Moore
P106	*Tabebuia rosea* (Bertol.) Bertero ex A.DC.
P107	*Tagetes erecta* L.
P108	*Tamarindus indica* L.
P109	*Tamilnadia uliginosa* (Retz.) Tirveng. & Sastre
P110	*Tecoma stans* (L.) Juss. ex Kunth
P111	*Thalia geniculata* L.
P112	*Thespesia populnea* (L.) Sol. ex Corrêa
P113	*Thunbergia grandiflora* (Roxb. ex Rottl.) Roxb.
P114	*Thunbergia laurifolia* Lindl.

Label	Plant Species
P115	*Turnera ulmifolia* L.
P116	Unknown
P117	Unknown11
P118	Unknown13
P119	Unknown6
P120	*Vitex negundo* L.
P121	*Wrightia religiosa* (Teijsm. & Binn.) Benth. ex Kurz
P122	*Xanthostemon chrysanthus* (F.Muell.) Benth.
P123	*Zephyranthes minuta* (Kunth) D.Dietr.
P124	*Zoysia japonica* Steud.
