# Supplementary material for: Habitat and landscape factors influence pollinators in a tropical megacity, Bangkok, Thailand
Source: PeerJ. 2018 Jul 20;6:e5335. doi: 10.7717/peerj.5335 (PMC6055598; doi:10.7717/peerj.5335)
Supplement: Supplemental Information 8 — None of our pollinator metrics showed a significant preference for one over the other. [file peerj-06-5335-s008.pdf]

Habitat and landscape factors influence pollinators in a tropical megacity, Bangkok, Thailand

Supplemental Table S4. Results from t-test analyses comparing pollinator richness and pollinator abundances at native and exotic plant species. None of our pollinator metrics showed a significant preference for one over the other.

	Native average	Exotic average	*t*-value	df	*P*
Total pollinator abundance	1.016	1.131	0.22	93	0.8
Total pollinator richness	1.657	1.472	0.79	86	0.4
Tetragonula stingless bee abundance	30.45	19.87	1.2	45	0.2
Apis honey bee abundance	9.213	15.979	1.3	92	0.2
A. cerana abundance	5.20	3.95	0.43	50	0.7
A. dorsata abundance	0.8509	3.868	1.4	62	0.2
A. florea abundance	3.162	8.161	1.5	69	0.1
Xylocopa carpenter bee abundance	0.5263	0.4200	0.3	76	0.8
Butterfly abundance	1.259	3.355	0.66	69	0.5
